# Pathology Influences Blood Pressure Change following Vagal Stimulation in an Animal Intubation Model

**DOI:** 10.1371/journal.pone.0069957

**Published:** 2013-08-21

**Authors:** Peter Jones, Laurent Guillaud, Christophe Desbois, Jean-Francois Benoist, Helene Combrisson, Stephane Dauger, Mark J. Peters

**Affiliations:** 1 Critical Care Group - Portex Unit, Institute of Child Health, University College London, United Kingdom; 2 Assistance Publique-Hôpitaux de Paris, Réanimation Pédiatrique, Hôpital Robert Debré, Paris, France; 3 Ecole Nationale Véterinaire d'Alfort, Maisons-Alfort, France; 4 Assistance Publique-Hôpitaux de Paris, Biochimie, Hôpital Robert Debré, Paris, France; 5 Université Paris Diderot, Sorbonne Paris Cité, Paris, France; Kaohsiung Chang Gung Memorial Hospital, Taiwan

## Abstract

**Purpose:**

The haemodynamic response to critical care intubation is influenced by the use of sedation and relaxant drugs and the activation of the vagal reflex. It has been hypothesized that different disease states may have a contrasting effect on the cardiovascular response to vagal stimulation. Our objective was to determine whether the blood pressure response to vagal stimulation was modified by endotoxaemia or hypovolaemia.

**Methods:**

New Zealand White rabbits were anaesthetised with urethane before tracheotomy. The exposed left Vagus nerve of randomised groups of control (n = 11), endotoxin (n = 11, 1 mg/kg), hypovolaemia 40% (n = 8) and hypovolaemia 20% (n = 8) rabbits were subjected to 10 Hz pulsed electrical stimulations of 25 s duration every 15 min. Haemodynamic parameters were recorded from a catheter in the right carotid artery connected to an iWorx monitor. Serum catecholamines were measured every 30 min using reverse-phase ion-pairing liquid chromatography. The change in blood pressure after vagal stimulation was compared to controls for one hour after the first death in the experimental groups.

**Results:**

29% of the rabbits died in the hypovolaemia 40% group and 27% in the endotoxin group. One rabbit died in the hypovolaemia 40% group before vagal stimulation and was excluded. Following electrical stimulation of the Vagus nerve there was a fall in blood pressure in control rabbits. Blood pressure was conserved in the hypovolaemic rabbits compared to controls (p<0.01). For the endotoxaemic rabbits, there was a non-significant trend for the mean blood pressure to decrease more than the controls. Serum catecholamines were significantly raised in both the hypovolaemic and endotoxaemic rabbits.

**Conclusions:**

Pathology may contribute to modifications in blood pressure when vagal activation occurs. Patients who are either already vasoconstricted, or not vasoplegic, may be less at risk from intubation-related vagally mediated reductions in blood pressure than those with vasodilatory pathologies.

## Introduction

The intubation of children during critical care illness can provoke alterations in both heart rate [1], [2], [3] and blood pressure [4], [5]. Heart rate generally falls during intubation due to the activation of the Vagus nerve, either by hypoxia in the aortic bodies or mechanical stimulation of the superior laryngeal nerve [3], [6]. Certain induction [7], [8] and muscle relaxant drugs [9] can also contribute to falls in heart rate.

Changes in blood pressure during intubation in critical care illness are more complex. In adults there is a tendency for the mean blood pressure to fall by 30 mmHg [10]. In contrast, an increased blood pressure has been reported during neonatal intubation despite significant decreases in heart rate [4], [5]. A possible explanation for these discordant observations is that different diseases may have different effects on haemodynamics during intubation. For example, sepsis reduces peripheral vasomotor tone [11] whereas hypovolaemia is associated with vasoconstriction [12]. Certain anaesthetic induction agents also modify vascular tone [13].

Our objective was to determine whether the blood pressure response to vagal stimulation was modified by endotoxaemia or hypovolaemia. Haemodynamic, biochemical and hormonal parameters were used to evaluate the effects of endotoxaemia and hypovolaemia on the rabbits.

## Methods

The study was approved by the ComEth Anses/ENVA/UPEC (Comité d'Ethique pour l'Expérimentation Animale, Maison-Alforts, FRANCE). All research animals were maintained under anaesthesia from the start of experimentation to the end when they were euthanised with a lethal dose of anaesthetic (5 g/kg urethane). Male New Zealand White (NZW) rabbits were obtained from Hypharm, La Corbière, 49450, Roussay, France. In line with recommendations, a maximum of 15 rabbits at a time were maintained in an aired pen 3 m by 4 m and 3 m high with a dense layer of straw and hay [14]. Food and water was provided *ad libitum*.

### Preparation of the rabbits

The rabbits were anaesthetised by the injection urethane in a marginal vein of an ear. 1.8 g/kg was used for rabbits weighing ≤1.8 kg and 2.0 g/kg for rabbits weighing >1.8 kg. The level of anaesthesia was monitored using paw and corneal reflexes. Urethane was chosen as an anaesthetic because of its single dose regimen and because it has previously been established to have minimal effect on haemodynamic parameters in anaesthetised rats [15]. Another advantage of using urethane was that the animals remained spontaneously breathing.

Sterile surgical conditions were used to make a midline incision in the anterior aspect of the neck. The trachea was opened before being intubated with a 7 cm plastic tube of with an internal diameter of 4.0 mm and 5.6 mm external diameter (Saint Gobain Verneret, La Mothe-aux-Aulnaires, F89120 Charny, France). The right carotid artery was cannulated with a 22gauge canula, and the left Vagus nerve was exposed. Continuous mean blood pressure readings were made using an iWorx® 214 monitor connected to the carotid artery catheter (iWorx Systems Inc, 62 Littleworth Raod, Dover NH 03820, USA). Arterial blood gas measurements were made using an i-STAT® system (Abbott Point of Care Inc., 400 College Road East, Princeton, NJ 08540, USA). The rabbits were entered into the experiment when baseline blood pressure had been stable for at least 15 minutes and arterial pH was between ≥7.35 and ≤7.45 (normal limits of rabbit pH 7.2–7.5 [16]).

### Conduct of the experiment

The rabbits were randomly assigned into four groups; control (11 rabbits), endotoxaemia 1 mg/kg (11 rabbits), hypovolaemia 20% reduction in blood volume (HV20%, 8 rabbits) and hypovolaemia 40%reduction in blood volume (HV40%, 8 rabbits).

Rabbits in the hypovolaemia groups were bled (estimated blood volume of 5.5 ml of blood per 100 g of body weight [17]) over a period of five minutes from the carotid artery catheter 20 minutes after baseline. The rabbits assigned to the endotoxaemia group were injected with 1 mg/kg of endotoxin (LPS from E.Coli B:055, Sigma-Aldrich, France) diluted at 1 mg/ml in 0.9% saline solution at a rate of 2 mg/5 minutes in the marginal vein of an ear.

### Blood Sampling and Biochemical Analysis

**Figure 1:**
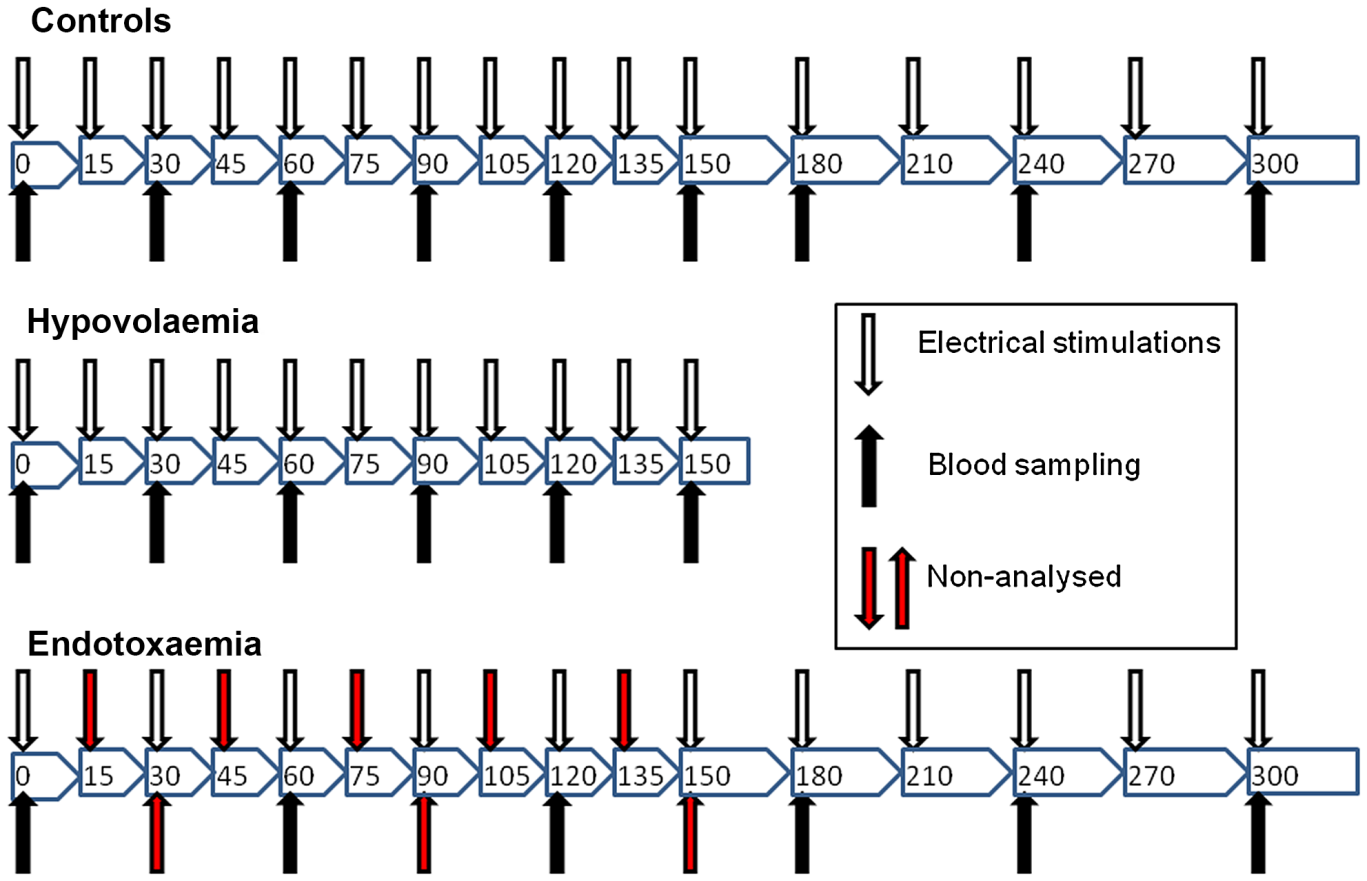
Gant chart showing schema of blood sampling and electrical stimulation. Control rabbits and the experimental groups all received the same number of stimulations and blood samples. Endotoxaemic rabbits had five stimulations and three blood samples that were not analysed, as according to protocol, so as to ensure identical experimental conditions for all groups.

Blood sampling was performed at the following times for all rabbits; baseline, then 30 minute intervals until 150 minutes. At 150 minutes the surviving hypovolaemic rabbits were euthanised. The control and endotoxaemic rabbits were then sampled at 180 minutes and then 60 minute intervals until 300 minutes, when the survivors were euthanised. The samples taken from the endotoxaemic rabbits at 30, 90 and 150 minutes were not analysed so as to ensure identical experimental conditions for all groups, see Figure 1.

Samples of 1.3 ml of blood were drawn from the carotid artery. Seventy microlitres were separated for blood gas (pH, PCO2, base excess, bicarbonates) and serum lactate (normal range 0.5–1.5 mmol/l [18]) analysis. A haematocrit (NZW normal range 31.3–43.3%, mean weight of rabbit 3.6 kg [19]) was determined by placing 0.1 ml of whole blood in a heparin coated plastic tube (100 µl safeCLINITUBES, Radiometer Medical AsP, Akandevej 21, 2700 Bronshoj, Denmark) which was centrifuged at 2000 revolutions/minutes for four minutes. The remaining 1.1 ml was transferred into an EDTA tube, shaken gently before centrifugation at 4000 rpm at a temperature of 4°C for 5 minutes. Thereafter, the plasma was drawn off and transferred directly for storage at −40°C in the dark for subsequent catecholamine analysis.

### Stimulation of the Vagus Nerve

The Vagus nerve was intermittently lifted free from other structures in the neck, laid across two stainless steel electrodes 5 mm apart and electrically stimulated using the iWorx system with a 3.0 mA current, 250 pulsations of 10 milliseconds at 10 Hz (duration of stimulation 25 seconds). A three-minute interval was left after taking blood before a vagal stimulation. Stimulations were made at baseline, then at 15 minute intervals for all rabbits until 180 minutes and then half-hourly for the control and endotoxaemic rabbits until 300 minutes, see Figure 1.

### Catecholamine analysis

All catecholamine analyses were performed at the Hôpital Robert Debré, Paris, France in accordance with the reverse-phase ion-pairing liquid chromatography methodology described by Candito *et al.* in 2002 [20]. Fifteen to 25 samples were batched for analysis. A negative control (acid), internal control (urine catecholamine) and standard control (quantified plasma catecholamine [Chromosystems Instruments and Chemicals GmbH, Heimburgstrasse 3, 81243 München, Germany]) were analysed in sequence before each batch.

Briefly, the plasma was removed from storage and thawed at room temperature. Six hundred microlitres was drawn from the plasma samples and 20 mg of alumina (aluminium oxide, Al_2_O_3_) and 100 µl of the internal control were added to the 600 µl of plasma. The alumina was washed three times before separation of the catecholamines from the alumina. Fifty microlitres of the supernatant were removed for injection through a 717 Waters auto-sampler onto a C18 Purospher (Merck) column (150×4 mm, 4 µm) equipped with a guard column. The aqueous isocratic mobile phase adjusted to pH 3.9 consisted of citric acid 20 mM, sodium acetate 50 mM, sodium heptane sulfonate 0.4 mM in methanol 10%. Mobile phase was pumped at a flow rate of 1 ml/min by a Waters Model 550 pump. Quantification of the catecholamines was carried out using an amperometric 2465 Waters detector set at 0.5 V. Millennium Waters Empower Chromatography software was used for all calculations.

### Presentation of results

The first death in each group was used *post hoc* as a measure of severity of pathology. Results are presented for one hour of monitoring from the vagal stimulation prior to the first death.

### Statistical analysis

Qualitative variables are described as numbers and percentages and quantitative variables as median [quartiles] or mean (standard deviation) according to their Gaussian distribution. An unpaired t-test, or a Mann-Whitney test, were used for continuous data according to their distribution. All statistical tests were 2-sided and the probability of a type 1 error (α) was determined at <0.05. All statistical tests were carried out using SPSS (version 19).

## Results

### Baseline Characteristics

Baseline characteristics of animals in each group were similar with the exception of adrenaline and noradrenaline which were significantly higher in the endotoxin group *versus* the control group (Table 1).

**Table 1:** Baseline characteristics of the rabbits.

	Control t = 0 (n = 11) (reference)	Endotoxin t = 0 (n = 11)	Hypovolaemia 20% t = 0 (n = 8)	Hypovolaemia 40% t = 0 (n = 7)
Weight (kg)	1.96 (1.79–2.13)	1.93 (1.76–2.10)	1.91 (1.71–2.11)	1.87 (1.70–2.04)
pH	7.39 (7.37–7.42)	7.39 (7.37–7.40)	7.39 (7.37–7.41)	7.38 (7.36–7.42)
Lactate (mmol/l)	2.9 [1.8–5.5]	2.3 [1.7–3.3]	3.6 [1.7–5.5]	3.0 [2.6–4.5]
Bicarbonate (mmol/l)	25.4 (22.6–28.0)	24.0 (21.7–24.5)	24.8 (24.5–27.3)	23.4 (22.5–24.3)
pCO2 (kPa)	5.5 (5.2–6.2)	5.1 (5.0–5.4)	5.5 (5.1–6.0)	5.4 (5.0–5.7)
Haemotocrit (%)	42 (37–45)	45 (42–46)	43 (40–48)	42 (39–47)
Adrenaline (nM)	2.0 [0–5.2]	7.9 [4.3–9.0] ¤	1.8 [0.6–5.8]	0.7 [0.5–2.6]
Noradrenaline (nM)	0.3 [0–1.6]	1.5 [0.6–2.4] *	0.4 [0–1.4]	0.4 [0–0.5]
Heart rate (bpm)	310 (278–323)	279 (267–303)	289 (270–297)	294 (264–311)
Pulse pressure (mmHg)	27 (20–31)	27 (23–29)	24 (19–28)	29 (19–32)
Blood pressure (mmHg)	77 (58–92)	72 (64–79)	63 (54–80)	73 (64–80)
Change in blood pressure after vagal stimulation(mmHg)	28 (21–37)	28 (18–31)	23 (14–29)	22 (17–30)

Interquartile ranges [IQR] and standard deviations are shown (SD) according to the distribution of the variables.

* p<0.05,

¤ p<0.01.

### Mortality and Presentation of the Results

One rabbit from the 40% hypovolaemia group died after bleeding and before vagal stimulation and was excluded from subsequent analysis. There were two deaths in the HV40% group (2/7, 29%) and three in the endotoxin group (3/11, 27%). No rabbits died in the HV20% group. The first death after vagal stimulation in the HV40% group occurred between +15 and +30 minutes from bleeding and between +240 and +270 minutes in the endotoxin group. According to protocol, the rabbits' parameters were analysed from +15 minutes in the hypovolaemia groups and +240 minutes for the endotoxin group for a period of one hour.

### Changes in Biochemical and Haematological Parameters in Hypovolaemia

The hypovolaemia groups showed decreases in pH, CO2, HCO3, haematocrit and pulse pressure, mean blood pressure and heart rate with a increases in adrenaline and lactates compared to the control group (Tables 1 and 2). These changes were proportional to severity of hypovolaemia.

**Table 2:** Changes in biochemistry, catecholamines and haematocrit in the hypovolaemic rabbits.

	Group	Time t = +15	Time t = +45	Time t = +75
Number of rabbits	Control	11	11	11
	HV 20%	8	8	8
	HV 40%	7	6 (1 death)	5 (2 deaths)
pH	Control (ref)	7.39 (7.37–7.41)	7.40 (7.37–7.41)	7.39 (7.37–7.41)
	HV 20%	7.34 (7.29–7.38)	7.35 (7.28–7.39)	7.34 [7.29–7.38)
	HV 40%	7.40 (7.33–7.43)	7.25 (7.1–7.3) ¤	7.30 (7.12–7.3) ¤
Lactate (mmol/l)	Control (ref)	3.3 [1.8–5.5]	3.5 [1.5–5.6]	4.2 [1.5–5.6]
	HV 20%	4.3 [1.3–6.7]	5.5 [2.0–8.8]	5.9 [2.0–9.8]
	HV 40%	5.5 [4.1–5.9]	9.2 [5.7–11.7] ¤	8.7 [6.0–16.1] *
Bicarbonate (mmol/l)	Control (ref)	26.6 (23.6–28.1)	25.3 (24.6–28.4)	25.4 (24.5–28.1)
	HV 20%	24.8 (21.4–26.4)	22.6 (21.6–23.9) ¤	22.2 (20.6–26.6)
	HV 40%	18.6 (17.9–22.1) ¤	16.5 (12.9–19.2) ¤	16.2 (12.2–18.4) ¤
pCO2 (kPa)	Control (ref)	6.0 (5.5–6.2)	5.8 (5.4–6.2)	5.8 (5.5–6.0)
	HV 20%	5.2 (5.0–6.2)	5.2 (4.9–6.0)	5.5 (5.1–6.4)
	HV 40%	4.5 (4.1–4.6) ¤	4.6 (4.4–7.3) *	4.5 (4.0–5.6)
Haematocrit (%)	Control (ref)	41 (37–47)	41 (37–45)	41 (36–44)
	HV 20%	38 (36–40)	37 (35–39)	36 (33–38) *
	HV 40%	36 (28–38) *	32 (28–34) ¤	32 (26–34) ¤
Adrenaline (nM)	Control (ref)	0.45 [0–2.4]	0 [0–0.55]	0 [0–0.55]
	HV 20%	0.83 [0.32–1.2]	0.14 [0–1.1]	0.62 [0.7–1.5]
	HV 40%	2.45 [0.43–13.9]	1.3 [0.36–22.4] *	1.34 [0–3.7]
Noradrenaline (nM)	Control (ref)	1.00 [0–3.2]	0.40 [0–2.1]	0 [0–1.9]
	HV 20%	2.42 [0.55–3.8]	1.50 [0.10–3.9]	1.79 [0.11–3.2]
	HV 40%	0.39 [0–1.9]	0.56 [0.37–7.7]	0.88 [0.38–3.6]

Interquartile ranges [IQR] and standard deviations are shown (SD) according to the distribution of the variables.

* p<0.05,

¤ p<0.01.

### Changes in Biochemical and Haematological Parameters in Endotoxaemia

**Figure 2:**
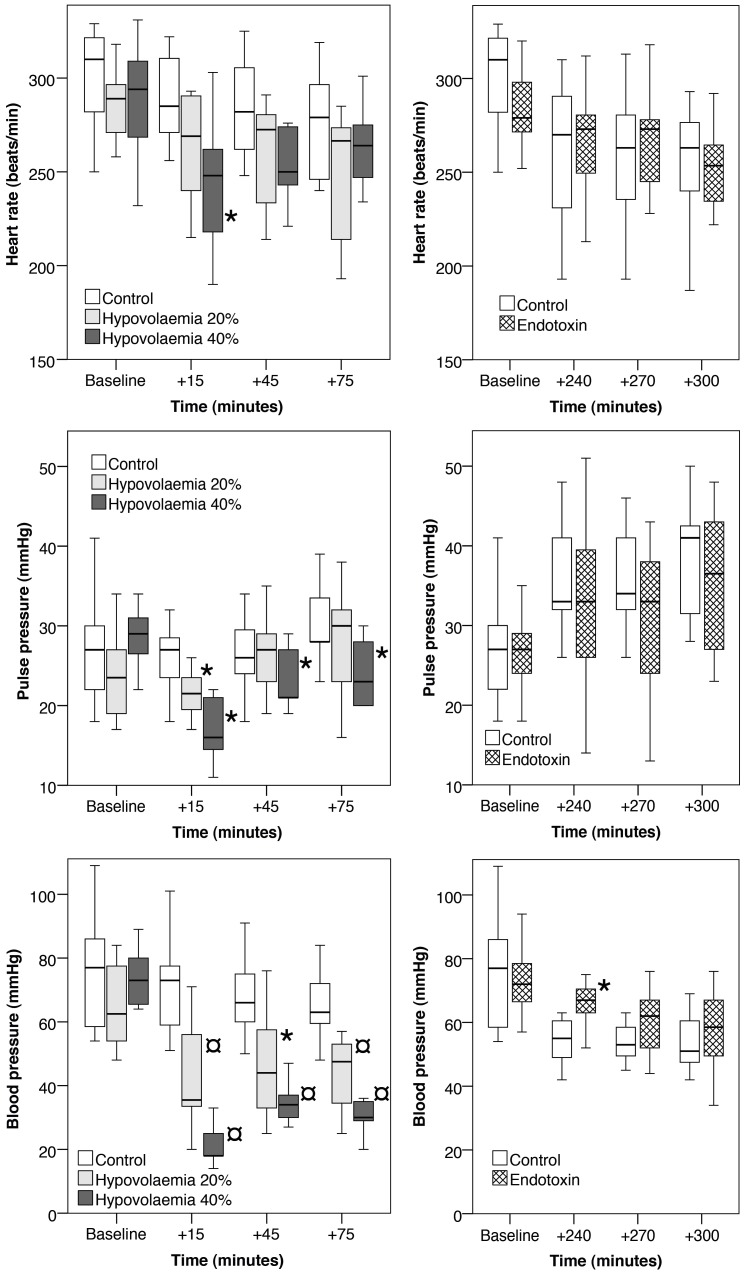
Changes in haemodynamic parameters for the control; hypovolaemia and endotoxaemia rabbits. The central bar is the mean, the box represents the inter-quartile range and the whiskers the range. Significant differences between the control and experimental groups of p<0.05 are noted by an ‘*’ and p<0.01 by an ‘¤’.

In the endotoxin group there were significant decreases in CO2 and bicarbonate with no change in pH, lactates and haematocrit and a significant increase in noradrenaline (Table 3). There were no changes in the haemodynamic parameters between control and endotoxaemic rabbits except a difference in blood pressure at +240 minutes (Figure 2).

**Table 3:** Changes in biochemistry, catecholamines and haematocrit in the endotoxin group.

		Time t = +240	Time t = +300
Number of rabbits	Control	11	11
	Endotoxin	11	8 (3 deaths)
pH	Control (ref)	7.35 (7.34–7.40)	7.36 (7.35–7.39)
	Endotoxin	7.35 (7.33–7.40)	7.33 (7.28–7.36)
Lactate (mmol/l)	Control (ref)	4.2 [2.0–5.7]	3.3 [1.8–5.6]
	Endotoxin	3.5 [2.1–5.1]	4.2 [1.8–5.1]
Bicarbonate (mmol/l)	Control (ref)	24.9 (23.9–26.4)	25.2 (23.5–27.4)
	Endotoxin	21.7 (18.4–22.4) ¤	21.0 (20.1–22.4) ¤
pCO2 (kPa)	Control (ref)	6.1 (5.7–6.5)	5.9 (5.6–6.2)
	Endotoxin	4.9 (4.6–5.4) ¤	5.2 (4.7–6.3)
Haematocrit (%)	Control (ref)	39 (37–43)	40 (37–43)
	Endotoxin	43 (39–44)	38 (38–41)
Adrenaline (nM)	Control (ref)	0.29 [0–0.73]	0.31 [0–1.1]
	Endotoxin	1.13 [0–3.5]	0.66 [0–2.8]
Noradrenaline (nM)	Control (ref)	0.75 [0–3.2]	0.36 [0–3.7]
	Endotoxin	3.45 [2.3–6.0] *	3.55 [2.4–5.0] ¤

Interquartile ranges [IQR] and standard deviations are shown (SD) according to the distribution of the variables.

* p<0.05,

¤ p<0.01.

### Changes in blood pressure following vagal stimulation

**Figure 3:**
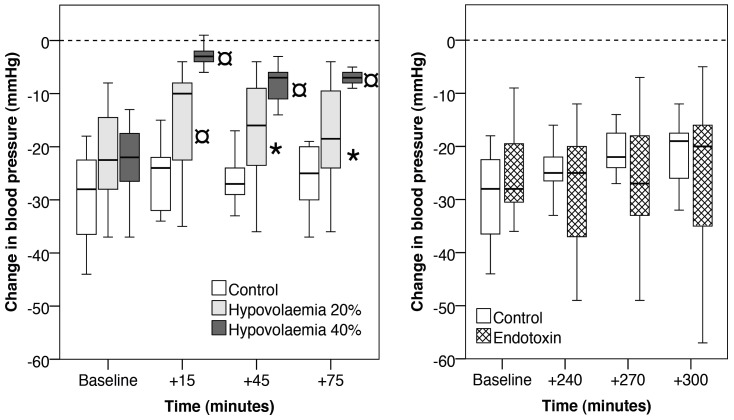
Change in mean blood pressure following vagal stimulation prior to the first death. Note, the times from the start of experimentation are different for the hypovolaemia (+15, +60 and +75 minutes) and endotoxin (+240 and +300 minutes) groups. The central bar is the mean, the box represents the inter-quartile range and the whiskers the range. Significant differences between the control and experimental groups of p<0.05 are noted by an ‘*’ and p<0.01 by an ‘¤’.

During electrical stimulation of the Vagus nerve there was significant, and dose dependent, maintenance of mean blood pressure in the hypovolaemic rabbits compared to controls (Figure 3). For the endotoxaemic rabbits, there was a non-significant trend for the mean blood pressure to decrease more than the controls (Figure 3).

## Discussion

We disproved our hypothesis. Following vagal stimulation the blood pressure of the hypovolaemic rabbits was significantly conserved when compared to controls. When compared to controls, endotoxaemic rabbits displayed a non-significant tendency towards a greater fall in blood pressure.

### Predicatable effets of hypovolaemia and endotoxaemia on haemodynamics

Classically the effect of hypovolaemic shock on haemodynamic integrity is biphasic and asymmetric. Mean blood pressure is maintained during the early compensatory Phase I by combination of increasing heart rate and vasoconstriction despite a fall in cardiac output [12], [21]. This is followed by a decompensatory Phase II which sees a fall in mean blood pressure and pre-load associated with a fall in vascular resistance despite maintenance of cardiac output [12], [21].

In a similarity with Phase I hypovolaemia, sepsis is associated with a reduction in pre-load and left ventricular stroke volume [22]. The difference with Phase I hypovolaemia is that whereas hypovolaemia is associated with vasoconstriction sepsis is associated vasodilatation [23].

### Observed effets of hypovolaemia and endotoxaemia in our model

In the hypovolaemia model, mean blood pressure fell markedly after bleeding (75 mmHg to 20 mmHg in the HV40% model) before increasing marginally (vasoconstrictive Phase I) to reach a plateau and then falling (vasodilatory Phase II) (Figure 2). Generally, the rabbits showed predictable haemodynamic changes with the exception of a fall in heart rate. This unexpected observation has previously been observed in hypovolaemic humans where reduced pre-load is compensated for by a prolongation of diastole which allows better ventricular filling [24]. Parallel dose-related changes in pH, bicarbonates, CO2 and lactates corroborated the haemodynamic alterations identified.

The effect of the endotoxin in our model is more difficult to interpret. Our rabbits did not demonstrate the decrease in blood pressure as early as 1–2 hr after injection that has been observed in other studies in rabbits (1 mg/kg) [25] and dogs (1.5 mg/kg) [26]. Neither was there a significant change in heart rate. Despite the relative paucity of haemodynamic changes, the mortality of the rabbits was similar to that observed in the HV40% model and the endotoxaemic rabbits displayed a significant compensated metabolic acidosis. It is possible that haemodynamic changes could have been recorded after 300 minutes. However, experimentation was terminated at 6 h because of concerns that the time taken to prepare (approximately 1 h) and monitor (300 min) the rabbits could exceed the anaesthetic effect and due to the uncertainly regarding the derangement of physiological homeostasis by a prolonged anaesthetic exposure. Another possible weakness of our model is perhaps demonstrated by the absence of difference between lactate levels between the endotoxaemic and control rabbits, as has been previously observed by Lobo *et al.* [25].

### Catecholamines

Raised levels of noradrenaline and adrenaline have previously been demonstrated in animal models of hypovolaemia [27] and sepsis [28]. The effect of these hormones is to result in vasoconstriction [29]. Despite the fact that both the hypovolaemic and endotoxaemic rabbits displayed raised levels of circulating catecholamines, only the hypovolaemic rabbits exhibited diminished pulse pressure, which is an indirect maker of vasoconstriction (Figure 2). This suggests that there was a relative failure of vasoconstriction in the endotoxaemic rabbits.

All four experimental groups displayed raised levels of baseline circulating catecholamines after surgical preparation and before vagal stimulations. These were possibly due to the stress of anaesthetic induction and/or preparation. Another possibility is that the rabbits were improperly anaesthetised despite each being tested for paw and corneal reflexes [30]. After the start of the vagal stimulations a decrease in catecholamines was observed in all groups (results not shown) with the exception of the endotoxaemic rabbits. This may be due to the influence of the neuro-inflammatory pathway [31]. Zhang *et al.* [32] have demonstrated diminished production of catecholamines after repeated vagal stimulation in shocked dogs.

### The effect of vagal stimulation on blood pressure

Vagal stimulations at baseline and in control rabbits reduced blood pressure in the order of 20 to 30 mmHg (Figure 3). After vagal stimulation in the hypovolaemic rabbits there was a surprising maintenance of blood pressure (Figure 3). Indeed, there was an association between lower blood pressure and maintenance of blood pressure after vagal stimulation (Figure 3). Some severely hypovolaemic/hypotensive rabbits increased their mean blood pressure following vagal stimulations when their baseline stimulations in the same rabbits had resulted in decreased blood pressure.

The endotoxaemic rabbits they had similar baseline blood pressure compared to control rabbits (Figure 2) but died at the same rate as the HV40% rabbits. However, following vagal stimulation the endotoxaemic rabbits and were less able to maintain blood pressure than the controls (Figure 3). One possible explanation for these dichotomous results is the different state of vascular reactivity; the hypovolaemic rabbits being vasoconstricted and endotoxaemic rabbits being vasodilated.

### Limitations of our model

The most appropriate animal model of human sepsis is subject to conjecture [33]. Our aim by using an intravenous bolus of endotoxin was to obtain maximum harmonisation of experimental conditions. However, in the clinical situation there is rarely an explosive release of cytokines but a more gradual inflammatory response. The understanding of the haemodynamic response to in our endotoxin model would perhaps been helped had we monitored markers of systemic inflammation, such as TNF and IL-1. This was not done due to concerns regarding the extra volume of sampled blood. We did not explore the possible effects of gram positive sepsis.

Clearly the haemodynamic response to intubation is a multi-factorial process with operator experience, method of intubation, sedation and relaxant drugs all potentially contributing to change in blood pressure. Inevitably, the more that the associated influences around intubation are controlled for in an experimental environment the less that the model resembles the clinical situation. However, our vagal stimulation model retains some relevance where vagal activation is frequent, such as during the intubation of children who have predominant parasympathetic balance in the early part of life [34]. The use of repeated vagal stimulations in the same animals further distorts the relevance to the clinical situation. The advantage of this methodology, however, is that the changes in blood pressure over time demonstrate the evolution of both disease models and the effect of the vagal stimulations.

### Conclusions

Pathology may contribute to modifications in blood pressure when vagal activation occurs. Patients who are either already vasoconstricted, or not vasoplegic, may be less at risk from intubation-related vagally mediated reductions in blood pressure than those with vasodilatory pathologies. Our results require confirmation in a model with a larger cohort where vascular resistance, ejection volume and cardiac output are measured.
